# Molecular detection of *Borrelia burgdorferi* sensu lato – An analytical comparison of real-time PCR protocols from five different Scandinavian laboratories

**DOI:** 10.1371/journal.pone.0185434

**Published:** 2017-09-22

**Authors:** Malin Lager, Maximilian Faller, Peter Wilhelmsson, Vivian Kjelland, Åshild Andreassen, Rimtas Dargis, Hanne Quarsten, Ram Dessau, Volker Fingerle, Gabriele Margos, Sølvi Noraas, Katharina Ornstein, Ann-Cathrine Petersson, Andreas Matussek, Per-Eric Lindgren, Anna J. Henningsson

**Affiliations:** 1 Laboratory Medicine, Region Jönköping County, Jönköping, Sweden; 2 Division of Medical Microbiology, Department of Clinical and Experimental Medicine, Linköping University, Linköping, Sweden; 3 German National Reference Centre for Borrelia, Bavarian Health and Food Safety Authority, Oberschleißheim, Germany; 4 Clinical Microbiology, Laboratory Medicine, Region Jönköping County, Sweden; 5 Faculty of Engineering and Science, Department of Natural Sciences, University of Agder, Kristiansand, Norway; 6 Research Unit, Hospital of Southern Norway Trust, Kristiansand, Norway; 7 Division of Infectious Disease Control, Department of Virology, Norwegian Institute of Public Health, Oslo, Norway; 8 Department of Clinical Microbiology, Slagelse Hospital, Slagelse, Denmark; 9 Department of Medical Microbiology, Hospital of Southern Norway Trust, Kristiansand, Norway; 10 Division of Medicine, Skånevård Kryh, Region Skåne, Sweden; 11 Division of Laboratory Medicine, Department of Clinical Microbiology, Lund, Sweden; 12 Karolinska University Laboratory, Stockholm, Sweden; 13 Division of Clinical Microbiology, Department of Laboratory Medicine, Karolinska Institutet, Karolinska University Hospital Huddinge, Stockholm, Sweden; University of Kentucky College of Medicine, UNITED STATES

## Abstract

**Introduction:**

Lyme borreliosis (LB) is the most common tick transmitted disease in Europe. The diagnosis of LB today is based on the patient´s medical history, clinical presentation and laboratory findings. The laboratory diagnostics are mainly based on antibody detection, but in certain conditions molecular detection by polymerase chain reaction (PCR) may serve as a complement.

**Aim:**

The purpose of this study was to evaluate the analytical sensitivity, analytical specificity and concordance of eight different real-time PCR methods at five laboratories in Sweden, Norway and Denmark.

**Method:**

Each participating laboratory was asked to analyse three different sets of samples (reference panels; all blinded) i) cDNA extracted and transcribed from water spiked with cultured *Borrelia* strains, ii) cerebrospinal fluid spiked with cultured *Borrelia* strains, and iii) DNA dilution series extracted from cultured *Borrelia* and relapsing fever strains. The results and the method descriptions of each laboratory were systematically evaluated.

**Results and conclusions:**

The analytical sensitivities and the concordance between the eight protocols were in general high. The concordance was especially high between the protocols using *16S* rRNA as the target gene, however, this concordance was mainly related to cDNA as the type of template. When comparing cDNA and DNA as the type of template the analytical sensitivity was in general higher for the protocols using DNA as template regardless of the use of target gene. The analytical specificity for all eight protocols was high. However, some protocols were not able to detect *Borrelia spielmanii*, *Borrelia lusitaniae* or *Borrelia japonica*.

## Introduction

Lyme borreliosis (LB) is the most common tick-borne disease in both Europe and Scandinavia, with large variation from 1/100,000 to >100/100,000 cases per year between different countries in Europe [1, 2]. The disease is caused by spirochetes belonging to the *Borrelia burgdorferi* sensu lato (s.l.) complex, and clinical manifestations of LB may include erythema migrans (EM), Lyme neuroborreliosis (LNB), acrodermatitis chronica atrophicans (ACA) and Lyme arthritis (LA) [3].

The diagnosis of LB is based on a combination of the patient´s medical history, clinical signs and symptoms and laboratory analyses. The microbiological analyses are mainly based on indirect detection of *B*. *burgdorferi* s.l. infection through antibody detection by enzyme-linked-immunosorbent assay (ELISA), which may be supplemented by immunoblot. Even though the ELISA method is widely used, it exhibits biological limitations due to delay of antibody formation [4], cross-reactivity [5, 6] and high seroprevalence in healthy populations in endemic areas [7–10]. Cultivation of the *Borrelia* spirochete is not used in clinical practice since it requires a long incubation time, is time consuming and laborious, has poor sensitivity in clinical samples (10–70%) and is susceptible to contamination [11, 12]. The need for a fast and reliable diagnostic tool is high for both patients and health care providers. Direct detection by PCR is a time efficient, reproducible, sensitive and specific method commonly used for detection of bacteria and viruses. Even though PCR is not suitable as a primary diagnostic tool for LB, probably due to the low numbers of spirochetes in most clinical cases, it may serve as a supplement to serology for certain conditions as well as in confirmation and genotyping of the infecting *Borrelia* spirochetes in suspected LB [11].

The clinical samples presenting the highest sensitivity of PCR for detection of *B*. *burgdorferi* s.l. are skin biopsies from patients with EM (36–88%) and ACA (54–100%) [11] as well as synovial fluid (SF) from LA patients (50–70%), while those with the lowest sensitivity are cerebrospinal fluid (CSF) (10–30%) [12, 13] and blood (10–20%) [11, 13].

PCR diagnosis of LB is based on the detection of one or more *B*. *burgdorferi* s.l. target genes. More than 20 target genes used for Borrelia detection (*e*.*g*., *16S* rRNA, *flaB*, *ospA* and 5S-23S intergenic spacer) have been published, but so far none of them has been widely implemented in laboratory practice. To the best of our knowledge no previous studies have compared different protocols on identical samples [14–16].

In 2011, a report regarding laboratory diagnostics of LB in Denmark, Finland, Norway and Sweden was published. A total of 43 laboratories participated in the survey, of which six offered detection of *Borrelia*-specific DNA by PCR. However, among these six laboratories, real-time PCR data was only available from five. Among a total of 582 samples extracted from CSF, skin biopsies and SF, 2.4% of the CSF samples were positive while 13% of the skin biopsies and SF samples generated positive results. This indicated that the most relevant material for PCR detection of *Borrelia* is skin biopsies and SF [17]. However, in this study only the rate of positivity was calculated and a comparison of specificity and sensitivity between the laboratories was not performed, which further supports the need for scrutiny of the PCR methods applied in LB diagnostics.

The objective of this study was to evaluate the analytical sensitivity and specificity together with the concordance of the real-time PCR methods currently in use in five laboratories in Scandinavia. The study includes an evaluation of the extraction protocols, PCR assays and the type of template (cDNA versus DNA) for the detection of *B*. *burgdorferi* s.l.

## Materials and methods

### Outline of the study

The study involved five laboratories (A-E) located in Scandinavia, including three clinical laboratories and two research laboratories, using eight different PCR protocols (1–8) (Table 1 and S1 Table). Two of the laboratories were located in Norway, two in Sweden and one in Denmark. Three blinded reference panels (described below) were sent to each laboratory, which analysed the samples according to their own routine real-time PCR protocol. The panels were also blinded for the coordinating laboratory. The results together with the method descriptions were reported to the Laboratory of Clinical Microbiology, Division of Medical Diagnostics, Region Jönköping County, Jönköping, Sweden (LCM, Sweden) for compilation.

**Table 1:** Summary of the PCR methods used for detection of *B*. *burgdorferi* s.l. at five laboratories (A-E) in Scandinavia. Protocol 1 used LUX technology and protocol 2–8 used TaqMan technology.

Laboratory	Protocol	Target gene	Name	Primer/Probe	Sekvens (5'–3')	Final concentration (μM)^1^	Fragment size (bp)	Instrument^2^	Reference
A	1	*16S* rRNA	B16S_FL	Forward	**gac tcG** TCA AGA CTG ACG CTG AGTC ^3^	0.200	131	CFX96	Wilhelmsson *et al*. 2010 [18]
			B16S_R	Reverse	GCA CAC TTA ACA CGT TAG CTT CGG TAC TAA C	0.200		(Bio-rad Laboratories Inc., Hercules, US)	
	2	*flaB*	flaBf	Forward	TCA AGA AAT AAT GST ATT AAT GCT GCTA A	0.600	98	ABI 7500	Jenkins *et al*. 2012 [19]
			flaBr	Reverse	CCA GCA GCA TCA TCA GAA GCT	0.600		(Applied Biosystems Inc.,	
			flaBmA	Probe	TGT ATC CAC TAG AAA GCT T	0.250		Carlsbad, US)	
			flaBm3B	Probe	TGT AAC CAC TAG AAA GCT T	0.250			
B	3	*16S* rRNA	16S F	Forward	GCT GTA AAC GAT GCA CAC TTG GT	0.500	69	LC480	Tsao *et al*. 2004 [16]
			16S R	Reverse	GGC GGC ACA CTT AAC ACG TTA G	0.500		(Roche Diagnostics,)	
			16S TM	Probe	TTC GGT ACT AAC TTT TAG TTA A	0.200		Basel, Switzerland)	
	4	*ospA*	ospA F	Forward	ATA TTT ATT GGG AAT AGG TCT AAT AT	0.500	137	LC480	Goosken *et al*. 2006 [20]
			ospA R	Reverse	CTT TGT CTT TTT CTT TRC TTA CAA G	0.500		(Roche Diagnostics)	
			osp TM	Probe	AAG CAA AAT GTT AGC AGC CTT GA	0.400			
C	5	*16S* rRNA	16SBOR_F	Forward	GGT CAA GAC TGA CGC TGA GTC A	0.400	136	MxPro 3005P Stratagene	Ornstein and Barbour 2006 [21]
			16SBOR_R	Reverse	GGC GGC ACA CTT AAC ACG TTA G	0.400		(Agilent Technologies Inc.,	
			16SBOR_P	Probe	TCT ACG CTG TAA ACG ATG CAC ACT TGG TG	0.100		Santa Clara, CA)	
D	6	*16S* rRNA	16SBOR-Fw	Forward	GGT CAA GAC TGA CGC TGA GTC A	0.400	136	CFX96	Ornstein and Barbour 2006 [21]
			16SBOR-Rev	Reverse	GGC GGC ACA CTT AAC ACG TTA G	0.400		(Bio-rad Laboratories Inc.)	
			16SBOR-P	Probe	TCT ACG CTG TAA ACG ATG CAC ACT TGG TG	0.100			
	7	*16S* rRNA	16SBor-sp-Fw	Forward	GCT GTA AAC GAT GCA CAC TTG GT	0.900	69	CFX96	Tsao *et al*. 2004 [16]
			16SBOR-Rev	Reverse	GGC GGC ACA CTT AAC ACG TTA G	0.900		(Bio-rad Laboratories Inc.)	
			16SBor-sp-P	Probe	TTC GGT ACT AAC TTT TAG TTA A	0.225			
E	8	*16S* rRNA	16S F	Forward	GCT GTA AAC GAT GCA CAC TTG GT	1.250	69	StepOnePlus	Tsao *et al*. 2004 [16]
			16S R	Reverse	GGC GGC ACA CTT AAC ACG TTA G	1.250		(Applied Biosystems Inc.)	
			LD-probe	Probe	TTC GGT ACT AAC TTT TAG TTA A	0.250			

^1^ Some changes have been made in the concentration of primer pairs and probes in comparison to the original protocols.

^2^ Extraction volumes and platforms: 1 and 2) 300 μL centrifuged CSF extracted on Biorobot EZ1 Advanced XL ((Qiagen, Hilden, Germany), 3 and 4) 200 μL centrifuged CSF extracted on Qiacube (Qiagen), 5) 500 μL uncentrifuged CSF extracted on NucliSENS® easyMag® (Biomerieux, Marcy-l'Étoile, France), and 6 and 7) 200 μL uncentrifuged CSF extracted on Biorobot EZ1 (Qiagen).

^3^ Bases in bold at the 5’ end of the B16S_FL primer correspond to additional bases added to create the hairpin loop structure.

### Reference panels for the molecular analysis and detection of *Borrelia burgdorferi* sensu lato

The reference panels of the study consisted of three different sample panels.

1. cDNA samples transcribed from total nucleic acid (NA) extracted from RNase-free water spiked with known concentrations of cultured *B*. *burgdorferi* s.l. (*B*. *afzelii* Lu81, *B*. *garinii* Lu59 and *B*. *burgdorferi* sensu stricto (s.s.) B31), including five negative controls (n = 20).
2. CSF samples spiked with known concentrations of *B*. *burgdorferi* s.l. bacteria (*B*. *afzelii* Lu81, *B*. *garinii* Lu59 and *B*. *burgdorferi* s.s. B31) (n = 15).
3. DNA samples of known concentration extracted from nine *B*. *burgdorferi* s.l. species (*B*. *burgdorferi* s.s. strains B31 and PBre, *B*. *afzelii* strains PKo and PVPM, *B*. *garinii* strains PBr, PHei, P WudII, Pref and PLa, *B*. *spielmanii* PSigII, *B*. *bavariensis* PBi, *B*. *bissetii* PGeb, *B*. *lusitaniae* Poti B2, *B*. *valaisiana* VS116 and *B*. *japonica*) and five specificity controls (*B*.*hermsii*, *B*. *miyamotoi*, *Treponema phagedenis* and *Leptospira*) (n = 95).

The sample materials were shipped on dry ice. The participants were asked to keep the samples for panels I and III at -20°C prior to analysis, the samples for panel II at -80°C and to avoid thawing and refreezing as this may affect the condition of the samples. Each sample was analysed in duplicate by the participants.

### Culture of *Borrelia burgdorferi* sensu lato strains (panels I and II)

All *B*. *burgdorferi* s.l. strains used for panels I and II were cultured at LCM, Sweden. The strains were kindly provided by Professor Sven Bergström, Umeå University, Sweden. One mL of each strain was cultured in 14 mL Barbour-Stoenner-Kelly (BSK) II medium [22] supplemented with 6% rabbit serum (Sigma Aldrich, St. Louis, Missouri, US). *B*. *afzelii* Lu81 was cultured at 35°C for 8 days, *B*. *garinii* Lu59 at 37°C for 7 days and *B*. *burgdorferi* s.s. B31 at 35°C for 6 days. The spirochetes were counted by phase-contrast microscopy. A 10-fold dilution series ranging from 2000 to 0.2 spirochetes μL^-1^ was prepared in RNase-free water (GE Healthcare Life Science, Chicago, Illinois, US) for each strain. These dilutions were used to spike samples in panels I and II. The samples were spiked and aliquoted immediately after the dilution series was prepared. The samples for panel I were extracted as described below, and the samples for panel II were placed at -80°C.

### Culture of *Borrelia burgdorferi* sensu lato strains and specificity controls (panels III)

All *B*. *burgdorferi* s.l. strains were isolated from patient materials except for *B*. *lusitaniae* Poti B2, *B*. *valaisiana* VS116 and *B*. *japonica*, which were all tick derived. The *B*. *burgdorferi* s.l. strains and *B*. *hermsii* were cultured at 33°C in modified Kelly-Pettenkofer (MKP) medium, harvested at a density of 10^7 cells mL^-1^ by centrifugation at 20,000 x *g* for 20 min, washed three times in 200 mL phosphate-buffered saline (PBS) pH 7.4 and resuspended in 200 mL PBS as previously described [23]. *Leptospira* strains were cultured at 28°C in Ellinghausen-McCullough-Johnson-Harris (EMJH) medium (Leptospira Medium Base EMJH BD, Difco^TM^ and Leptospira Enrichment EMJH Difco^TM^, New Jersey, USA) as described before [24]. *Treponema phagedenis* was grown in Fluid Thioglycollate Medium (FTM) (BioMerieux, Marcy l'Etoile, France) enriched with 10% rabbit serum (C. C. Pro GmbH, Germany) at 37°C. *B*. *miyamotoi* was grown in MKP medium with 50% human serum in a 6% CO_2_ atmosphere as previously described [25]. *Leptospira* strains and *T*. *phagedenis* were harvested at a density of 10^7 cells mL^-1^ as described in the text above.

### Extraction and reverse-transcription of nucleic acid (panel I)

A total of 5 μL of each dilution of each strain was used to spike 400 μL RNase-free water (GE Healthcare Life), resulting in a final concentration ranging from 10^4 to 10^0 spirochetes per sample. Total NA with no DNase treatment, was extracted at LCM, Sweden using a MagAttract® RNA Tissue Mini M48 kit (Qiagen, Hilden, Germany) and a BioRobot M48 Workstation (Qiagen) according to the manufacturer’s instructions with an insert volume of 400 μL. The total NA was eluted in a volume of 50 μL RNAse-free water (GE Healthcare Life). Reverse-transcribed NA (RTNA) synthesis was performed by using an Illustra ™ Ready-to-Go RT-PCR beads kit (GE Healthcare, Amersham, Place, UK). Fifteen μL of the extracted NA was incubated with 10 μL (0.25 μg μL^-1^) random hexamer primers (pd(N)_6_) at 97°C for 5 min. The beads were dissolved by adding 25 μL RNAse-free water (GE Healthcare Life), transferred to the NA/primer solution and incubated at 42°C for 30 min followed by 95°C for 5 min, resulting in a final volume of 50 μL. The RTNA was stored at -20°C. The panel also included five negative controls (three containing RNase-free water (GE Healthcare Life) and two containing DNA purified from *Escherichia coli*). Since each sample was eluted in 50 μL, referring to 100% of the sample in this study, and 15 μL per sample was used for the reverse-transcription of nucleic acid, the amount of sample used was approximately two third of the total sample volume. This means that each laboratory in panel I, but also in and II for laboratory A, has detected approximately 66.6% of the initial amount (10^4–10^0) of *Borrelia* spirochetes per sample (with the assumption of 100% NA extraction) instead of 100% which would have been the case if the reverse-transcription of nucleic acid had been done in triplicates using the total amount of the eluted sample material. The cDNA was pooled and aliquoted. A total of 50 μL per sample was sent out to the participating laboratories for amplification. Each laboratory was asked to use 5 μL per reaction.

### Preparation of samples for nucleic acid extraction from cerebrospinal fluid (panel II)

Five μL of each dilution of each strain (same dilution series as in panel I) was used to spike 1 mL of CSF, resulting in a final concentration ranging from 10^4 to 10^0 spirochetes mL^-1^. The CSF used was obtained from the Clinical Chemistry Laboratory, Division of Medical Diagnostics, Region Jönköping County, Jönköping, Sweden and consisted of pooled samples from patients without CSF pleocytosis and without clinically suspected LNB. All the patients were sampled for issues other than LNB (i.e. there were no clinical suspicions of LNB) and the samples were anonymized before use. The spiked CSF samples were stored at -80°C until transportation to the participating laboratories. Each laboratory was asked to extract total NA with no DNase treatment or DNA and analyse 5 μL per reaction by PCR according to their own protocol(s).

### Preparation of DNA samples from cultured *Borrelia* species (panel III)

A total of 95 samples containing DNA of known concentration extracted from 15 cultured *B*. *burgdorferi* s.l. strains (*B*. *burgdorferi* s.s. strains B31 and PBre, *B*. *afzelii* strains PKo and PVPM, *B*. *garinii* strains PBr, PHei, P WudII, Pref and PLa, *B*. *spielmanii* PSigII, *B*. *bavariensis* PBi, *B*. *bissetii* PGeb, *B*. *lusitaniae* Poti B2, *B*. *valaisiana* VS116 and *B*. *japonica*) were analysed by the five participating laboratories. The panel also included five specificity controls containing two relapsing fever strains (*B*. *hermsii* and *B*. *miyamotoi*), *Treponema phagedenis* and *Leptospira*, spirochetes closely related to *B*. *burgdorferi* s.l., in a final concentration of 2000 spirochetes μL^-1^.

Extraction of DNA was performed using a Maxwell® 16 LEV Blood DNA Kit (Promega Corporation, Madison, USA) on the Maxwell® 16 Instrument (Promega) as recommended by the manufacturer. DNA concentration was measured by NanoDrop™ 1000 Spectrophotometer (Thermo Scientific, Massachusetts, USA), and 15 mL of a solution containing 2000 organisms μL^-1^ was produced by adding 0.01 M Tris-buffer pH 8.0 molecular biology grade (AppliChem Panreac, Darmstadt, Germany). A 10-fold dilution series corresponding to 200–0.02 *Borrelia* spirochetes μL^-1^ was produced, and aliquots of 50 μL per dilution were stored frozen at -80°C until transportation to the participating laboratories.

### Detection of *Borrelia burgdorferi* sensu lato by real-time PCR at the participating laboratories

The samples in panels I-III were analysed by the participating laboratories according to the protocols presented in Table 1 and S1 Table. Each laboratory based their protocol on real-time PCR, with seven of eight protocols using TaqMan technology, and one of the eight used LUX technology. Seven of eight protocols detected the chromosomal target genes *16S* rRNA (n = 6) and *flaB* (n = 1) while one detected the plasmid target gene *ospA*. Three of eight protocols (protocols 3, 7 and 8) were based on Tsao *et al*. 2004 [16] and two of eight protocols (protocols 5 and 6) were based on Ornstein *et al*. 2006 [21] (Table 1). The protocols detecting the target gene *16S* rRNA (protocols 1, 3 and 5–8) are further referred to as *16S* rRNA PCR protocols, and the protocols detecting the target genes *ospA* and *flaB* (protocols 2 and 4) are further referred to as non-*16S* rRNA PCR protocols.

### Statistics

The analytical sensitivity was assessed for protocols 1–8 as well as for the *16S* rRNA PCR protocols in panels I–III using dilutions of cultured *Borrelia* species. Analytical specificity was assessed using controls spiked with *E*. *coli* and RNase-free water as well as two relapsing fever strains (*B*. *hermsii* and *B*. *miyamotoi*), *Treponema phagedenis* and *Leptospira*. The R-software was used for calculation of the 95% binomial confidence interval (CI) using the command binom.confint in package binom, choosing the Wilson method, which is a choice when CIs are close to the limits of zero or one.

## Results

### Real-time PCR of cDNA from spiked water samples (panel I)

The concentrations given as unit mL^-1^ in the cDNA panel I refer to the initial concentration of spirochetes prior to total NA extraction. As well, the amount of cDNA of each sample will be influenced by the level of mRNA expression of the target genes in the strains used. The results presented full concordance between the eight protocols down to 10^3 spirochetes mL^-1^ for *B*. *afzelii* Lu81 and *B*. *burgdorferi* s.s. B31 and full concordance as far as 10^1 spirochetes mL^-1^ for *B*. *garinii* Lu59 (Table 2 and S2 Table). At target concentration of 10^2 spirochetes mL^-1^ or below the ability to detect *Borrelia* cDNA varied between the protocols, especially for the non-*16S* rRNA PCR protocols, which had lower analytical sensitivity for *B*. *afzelii* Lu81 and *B*. *burgdorferi* s.s. B31. However, the *16S* rRNA PCR protocols showed full concordance between 10^4 and 10^1 spirochetes mL^-1^ for all the three *Borrelia* genospecies (Table 2 and S2 Table). At a concentration of 10^0 spirochetes mL^-1^, only one sporadic positive result was found, as would be expected. All negative controls containing RNase-free water (GE Healthcare Life) or *E*. *coli* (n = 40) were correctly identified as negative by all protocols, except for protocol 8 which gave a positive result for one of the negative controls (Table 2 and S2 Table). Thus, the analytical specificity of the PCR protocols taken together with five samples each was 95.7% (95% CI 87–100%).

**Table 2:** Results from panel I consisting of cDNA transcribed from total nucleic acid extracted from RNase-free water spiked with different concentrations of *B*. *afzelii* Lu81, *B*. *garinii* Lu59 and *B*. *burgdorferi* s.s. B31.

		Laboratory A		Laboratory B		Laboratory C	Laboratory D		Laboratory E
		*16S* rRNA	*flaB*	*16S* rRNA	*ospA*	*16S* rRNA	*16S* rRNA	*16S* rRNA	*16S* rRNA
Strain	Concentration^4^^,^^5^	Protocol 1	Protocol 2	Protocol 3	Protocol 4	Protocol 5	Protocol 6	Protocol 7	Protocol 8
*B*. *afzelii* Lu81	10^4	+	+	+	+	+	+	+	+
	10^3	+	+	+	+	+	+	+	+
	10^2	+	-	+	+	+	+	+	+
	10^1	+	-	+	-	+	+	+	+
	10^0	-	-	-	-	-	-	-	-
*B*. *garinii* Lu59	10^4	+	+	+	+	+	+	+	+
	10^3	+	+	+	+	+	+	+	+
	10^2	+	+	+	+	+	+	+	+
	10^1	+	+	+	+	+	+	+	+
	10^0	-	-	-	-	-	-	-	-
*B*. *burgdorferi* s.s. B31	10^4	+	+	+	+	+	+	+	+
	10^3	+	+	+	+	+	+	+	+
	10^2	+	-	+	-	+	+	+	+
	10^1	+	-	+	-	+	+	+	+
	10^0	+	-	-	-	-	-	-	-
*E*.*coli* J1	50	-	-	-	-	-	-	-	-
	50	-	-	-	-	-	-	-	-
RNase-free water		-	-	-	-	-	-	-	+
		-	-	-	-	-	-	-	-
		-	-	-	-	-	-	-	-

^4^ The unit for the *Borrelia* strains is spirochetes mL^-1^ while the unit for *E*. *coli* is ng μL^-1^.

^5^ The unit mL^-1^ in panel I corresponds to the original concentration prior to extraction, assuming 100% exchange, and not the expected concentration after cDNA synthesis.

### Real-time PCR of spiked cerebrospinal fluid samples (panel II)

The results from the seven protocols (panel II was not analysed by protocol 8 due to lack of resources) presented full concordance down to 10^3 spirochetes mL^-1^ for *B*. *afzelii* Lu81 and *B*. *burgdorferi* s.s. B31 and full concordance down to 10^2 spirochetes mL^-1^ for *B*. *garinii* Lu59 (Table 3 and S3 Table). The unit mL^-1^ in panel II refers to the original concentration prior to extraction, assuming 100% exchange and the use of the entire sample volume (1 mL). At target concentrations of 10^2 spirochetes mL^-1^ or below the ability to detect *Borrelia* cDNA or DNA varied between the protocols, especially for the two protocols extracting total NA (Laboratory A). However, the protocols extracting DNA showed full concordance down to 10^2 spirochetes mL^-1^ for all three *Borrelia* genospecies (Table 3 and S3 Table).

**Table 3:** Results from panel II consisting of cerebrospinal fluid spiked with different concentrations of *B*. *afzelii* Lu81, *B*. *garinii* Lu59 and *B*. *burgdorferi* s.s. B31. Protocol 8 was excluded due to lack of resources.

		Laboratory A		Laboratory B		Laboratory C	Laboratory D	
		cDNA	cDNA	DNA	DNA	DNA	DNA	DNA
Strain	Concentration^6^^,^ ^7^	Protocol 1	Protocol 2	Protocol 3	Protocol 4	Protocol 5	Protocol 6	Protocol 7
*B*. *afzelii* Lu81	10^4	+	+	+	+	+	+	+
	10^3	+	+	+	+	+	+	+
	10^2	-	-	+	+	+	+	+
	10^1	-	-	+	+	-	-	-
	10^0	-	-	-	-	-	-	-
*B*. *garinii* Lu59	10^4	+	+	+	+	+	+	+
	10^3	+	+	+	+	+	+	+
	10^2	+	+	+	+	+	+	+
	10^1	-	-	-	-	+	+	+
	10^0	-	-	+	+	-	+	+
*B*. *burgdorferi* s.s. B31	10^4	+	+	+	+	+	+	+
	10^3	+	+	+	+	+	+	+
	10^2	-	-	+	+	+	+	+
	10^1	-	-	-	-	-	-	-
	10^0	-	-	-	-	-	-	-

^6^ The unit for the *Borrelia* strains is spirochetes mL^-1^.

^7^ The unit mL^-1^ in panel II corresponds to the original concentration prior to extraction, assuming 100% exchange.

### Real-time PCR of samples with known concentration of DNA (panel III)

All eight protocols found concordant positive results down to 2 spirochetes μL^-1^ for the strains *B*. *burgdorferi* s.s. Pbre; *B*. *afzelii* PKo and PVPM; *B*. *garinii* PBr, PHei, PWudII, PRef and Pla; *B*. *bavariensis* Pbi; *B*. *bissetii* PGeb; and *B*. *valaisiana* VS116 (Table 4 and S4 Table). For *B*. *burgdorferi* s.s. B31 the protocols found concordant positive results down to >1 spirochete μL^-1^. The sample containing *B*. *spielmanii* PSigII showed concordant positive results down to 2 spirochetes μL^-1^ for all protocols except for one non-*16S* rRNA PCR protocol (protocol 4). The *B*. *lusitaniae* Poti B2 samples yielded concordant positive results down to 2 spirochetes μL^-1^ for all protocols except for the two used by Laboratory B (protocol 3 and 4), which did not detect it. *B*. *japonica* yielded concordant positive results down to 20 spirochetes μL^-1^ for three of the *16S* rRNA PCR protocols (protocol 1, 5 and 6) and negative results at all dilutions for the remaining protocols. *B*. *hermsii* yielded positive results for 2000 spirochetes μL^-1^ in all protocols except for the non-*16S* rRNA PCR protocols (protocols 2 and 4). *B*. *miyamotoi* yielded positive results for 2000 spirochetes μL^-1^ for all protocols except for one of the non-*16S* rRNA PCR protocols (protocol 4). Twenty-three of twenty-four samples spiked with *Leptospira* or *T*. *phagedenis*, were negative. The analytical specificity of the PCR protocols taken together was 96% (95% CI 80–99%).

**Table 4:** Results from panel III consisting of DNA extracted from 15 *Borrelia* strains and five specificity controls containing two relapsing fever strains (*B*. *hermsii* and *B*. *miyamotoi*), *Treponema phagedenis* and *Leptospira*.

		Laboratory A		Laboratory B		Laboratory C	Laboratory D		Laboratory E
Strains	Concentration^8^	Protocol 1	Protocol 2	Protocol 3	Protocol 4	Protocol 5	Protocol 6	Protocol 7	Protocol 8
*B*. *burgdorferi* s.s. B31	10^4–10^0	+	+	+	+	+	+	+	+
	10^-1	-	-	-	+	+	+	-	+
*B*. *burgdorferi* s.s. PBre	10^4–10^1	+	+	+	+	+	+	+	+
	10^0	-	+	+	+	+	-	+	+
	10^-1	-	-	-	-	+	-	-	-
*B*. *afzelii* PKo	10^4–10^1	+	+	+	+	+	+	+	+
	10^0	-	+	+	+	+	+	+	+
	10^-1	-	-	+	+	+	-	+	-
*B*. *afzelii* PVPM	10^4–10^1	+	+	+	+	+	+	+	+
	10^0	-	+	+	+	+	+	+	+
	10^-1	-	+	+	+	+	-	-	-
*B*. *garinii* PBr	10^4–10^1	+	+	+	+	+	+	+	+
	10^0	+	+	+	+	+	+	-	+
* *	10^-1	-	-	+	+	+	+	-	-
*B*. *garinii* PHei	10^4–10^1	+	+	+	+	+	+	+	+
	10^0	-	-	+	+	+	+	+	+
* *	10^-1	-	-	-	-	-	-	-	-
*B*. *garinii* PWudll	10^4–10^1	+	+	+	+	+	+	+	+
	10^0	-	-	+	+	+	+	+	+
* *	10^-1	-	-	+	+	+	-	-	-
*B*. *garinii* PRef	10^4–10^1	+	+	+	+	+	+	+	+
	10^0	-	+	+	+	+	+	+	+
* *	10^-1	-	-	-	+	-	+	+	+
*B*. *garinii* PLa	10^4–10^1	+	+	+	+	+	+	+	+
	10^0	+	+	+	-	+	+	-	+
* *	10^-1	-	-	-	-	-	+	-	+
*B*. *spielmanii* PSigII	10^4	+	+	+	-	+	+	+	+
	10^3	+	+	+	-	+	+	+	+
	10^2	+	+	+	-	+	+	+	+
	10^1	+	+	+	-	+	+	+	+
	10^0	-	-	-	-	+	+	+	+
* *	10^-1	-	-	-	-	-	-	-	-
*B*. *bavariensis* PBi	10^4–10^1	+	+	+	+	+	+	+	+
	10^0	-	+	+	+	+	+	+	-
	10^-1	-	-	-	-	-	-	-	-
*B*. *bissetii* PGeb	10^4–10^1	+	+	+	+	+	+	+	+
	10^0	-	+	+	+	+	-	+	+
* *	10^-1	-	-	-	+	+	-	-	+
*B*. *lusitaniae* Poti B2	10^4	+	+	-	-	+	+	+	+
	10^3	+	+	-	-	+	+	+	+
	10^2	+	+	-	-	+	+	+	+
	10^1	+	+	-	-	+	+	+	+
	10^0	-	+	-	-	+	+	+	+
* *	10^-1	-	-	-	-	+	-	+	+
*B*. *valaisiana* VS116	10^4–10^1	+	+	+	+	+	+	+	+
	10^0	-	+	+	+	+	+	+	+
* *	10^-1	-	-	+	+	-	+	-	-
*B*. *japonica*	10^4	+	-	-	-	+	+	-	-
	10^3	+	-	-	-	+	+	-	-
	10^2	+	-	-	-	+	+	-	-
	10^1	-	-	-	-	+	+	-	-
	10^0	-	-	-	-	+	-	-	-
* *	10^-1	-	-	-	-	-	-	-	-
*B*. *hermsii*	10^4	+	-	+	-	+	+	+	+
*B*. *miyamotoi*	10^4	+	+	+	-	+	+	+	+
*T*. *phagedenis*	10^4	-	-	-	-	-	-	-	-
*Leptospira*	10^4	-	-	-	-	+	-	-	-
*Leptospira*	10^4	-	-	-	-	-	-	-	-

^8^The amount of *Borrelia* spirochetes in 5 μL.

## Discussion

In this study we compared analytical sensitivity, analytical specificity and concordance between eight protocols for detecting *B*. *burgdorferi* s.l. by real-time PCR assays at five laboratories in Scandinavia. The concordance and analytical sensitivity between the protocols is generally high. However, the results demonstrate the importance of the choice of target gene as well as type of template (DNA/cDNA), especially regarding *16S* rRNA in relation to cDNA. A previous study including participants from 18 countries comparing different reverse transcriptase (RT)-PCR protocols for detection of tick-borne encephalitis virus (TBEV) showed that only 2 of 23 participants correctly identified all samples in the study, which concluded that there is a need for improvement in the sensitivity and specificity of molecular assays for the virus [26].

PCR may be a complementary diagnostic tool in spinal fluid from LNB patients in early disease when antibodies are not yet developed [11]. The method could be applicable in situations when the serological method is unable to distinguish between an acute and previous infection, similar to that in skin biopsies from EM where active and viable *Borrelia* spirochetes persist in IgG positive patients or in LA patients where DNA from dead bacteria may persist for months in synovial fluid of IgG positive patients [27]. However, clear recommendations for clinical use of PCR have not yet been proposed. Although PCR has high analytical sensitivity, bacterial DNA may be detected only if this is present in the patient material in sufficient concentration; thus, the clinical sensitivity has been found to vary. Hence, optimization of the methods is crucial. As the *B*. *burgdorferi* s.l. spirochetes can be found in very low numbers, the method is of limited value as a positive result is rare due to lack of bacteria in the sample, and a negative result cannot exclude infection. To improve both the analytical sensitivity and specificity of the PCR methods there are several aspects that are important to take into consideration, *e*.*g*. the target gene, the method of extraction, the type of template (total NA/cDNA/DNA), the primer and probe sequences, the PCR conditions and the thermocycler [11]. For instance, it is essential to use a target gene that is genetically stable [28], since loss or alteration of the target sequence may lead to loss of reactivity.

The results from panel I (Table 2 and S2 Table) in the present study demonstrate the importance of selecting a target gene in correlation with cDNA as the type of template. Laboratory A (protocols 1 and 2) and laboratory B (protocols 3 and 4) used two different target genes; *16S* rRNA versus *flab* and *16S* rRNA versus *ospA*, respectively, for detection of *B*. *burgdorferi* s.l. The results showed different detection limits between the protocols in favor of the *16S* rRNA PCR protocols when using cDNA as the type of template. Previous studies have shown that the heterogeneous plasmid target *ospA* is present in multiple copies in each bacterium, indicating that the sensitivity is higher than for the single-copy chromosomal target gene *16S* rRNA [29, 30]. However, if the variability of existing copies of the gene within the *Borrelia* genus is high one must not be misled to think that the sample contains a higher number of spirochetes then it actually does. In clinical samples this may be a pitfall and therefore, in cases were quantification of the *Borrelia* spirochetes is of interest, it is preferable to use a gene with a consistent copy number in the bacteria genome. The results from panel I consisting of cDNA as type of template demonstrate higher analytical sensitivity for the *16S* rRNA PCR protocols compared to the non-*16S* rRNA PCR protocols in combination with cDNA. This finding may be explained by the fact that since the rRNA is one of the most abundant molecules in the bacterial cell, there is potential for lowering the detection limit by more than one order of magnitude compared to genomic or plasmid DNA targets. Previous studies have shown that *16S* rRNA is more sensitive than assays based on the plasmid gene *ospA* [21]. However, there are some drawbacks to using RNA instead of DNA, such as additional cost in reagents and labour as well as high susceptibility of RNA to degradation and nucleases during extraction and storage. Despite the differences in detection limit between the non-*16S* rRNA PCR protocols, the concordance between the *16S* rRNA PCR protocols was high, wherein protocols 5 and 6 according to Ornstein *et al*. 2006 [20] and protocols 3, 7 and 8 according to Tsao *et al*. 2004 [16] each used the same set of primer pairs and probes. However, based on the use of cDNA, we can argue only that cDNA from cultured *Borrelia* is more reliably detected by the *16S* rRNA PCR protocols. The analytical sensitivity in terms of detectable genome copies remains unclear. In this context it would have been of high interest to establish the analytical sensitivity in the same type of samples but without cDNA transcription since the possible positive effect of the initial cDNA transcription might have become obvious. However, this could not be performed, since it was preferable to keep the study less laborious and less expensive for each participating part in order to make it feasible for as many laboratories as possible to participate in the study.

However, the project was set up so that we could conclude regarding this issue in panel III, containing DNA samples at different concentrations analyzed by the five participating laboratories. In panel III we saw that the analytical sensitivity is high and not affected by the choice of target gene, unlike in panel I where we find an increase sensitivity for the PCR protocols based on the target gene *16S* rRNA and when cDNA was used as the type of template. We are convinced that the same conclusions would have been made if we had extracted and analyzed the samples in panel I for DNA and compared it with the results generated by analysis of cDNA.

Since no quantitative PCR was performed the exact yield of total NA after extraction and cDNA after transcription is unknown, which may be considered a weakness in panel I as well as for protocols 1 and 2 in panel II. However, since most of the protocols were able to detect down to 10^1 spirochetes per mL^-1^, we assume that the yield was acceptable.

Panel II (Table 3 and S3 Table) demonstrates the importance of selecting the type of template in relation to the target gene, since the PCR results are highly dependent on this. Extracting total NA followed by cDNA-synthesis will theoretically result in higher numbers of targets per cell since the extraction from each bacterium will result in a high amount of specific RNA molecules together with DNA. Each sample will contain a mix of reverse-transcribed NA and extracted DNA since no DNase treatment was performed. When comparing the results generated from the different protocols we observed that the limit of detection (LOD) for the samples tested was the same within each laboratory regardless of type of target gene and that the LOD varies between the different laboratories depending on type of template. However, the analytical sensitivity in panel II was higher for the protocols analysing DNA compared to the protocols analysing cDNA, where the analytical sensitivity was lower. This strongly indicates the importance of pre-analytical factors other than extraction and type of template, such as sample volume, centrifugation of sample versus no centrifugation of sample, centrifugation speed and time, but also the use of fresh versus frozen samples, which may influence the analytical sensitivity and specificity. These parameters would be highly interesting to investigate in a further study. A comparison between the *16S* PCR protocols (excluding protocol 1 that extracted total NA), wherein protocols 5 and 6 according to Ornstein *et al*. 2006 [20] and protocols 3 and 7 according to Tsao *et al*. 2004 [16] each used the same set of primer pairs and probes, presented full concordance down to at least 10^2 spirochetes mL^-1^. However, protocol 3 was not able to detect the *B*. *garinii* Lu59 strain in the 10^1 spirochetes mL^-1^ dilution, but it was able to detect 10^0 spirochetes mL^-1^. This may be a result of the high dilution in 10^1–10^0 spirochetes mL^-1^, which may affect the reproducibility. However, it may also be a result of sample mix-up or contamination since the results from the *16S* rRNA PCR protocol (protocol 3) were reproducible for *ospA* (protocol 4).

In panel III (Table 4 and S4 Table), containing extracted DNA from nine *Borrelia* species, the analytical sensitivity of the different target genes within any single laboratory (*16S* rRNA versus *flaB* or *16S* rRNA versus *ospA*) was in generally high and comparable. However, we did not see any relation between DNA and any specific target gene like in panel I. Protocols 1, 5 and 6 were designed in silico to detect a segment of the *16S* rRNA gene DNA sequence present in practically all species of borreliae including *B*. *afzelii*, *B*. *garinii*, *B*. *burgdorferi s*.*s*., *B*. *valaisiana*, *B*. *lusitaniae*, *B*. *spielmanii*, *B*. *miyamotoi* and *B*. *hermsii*. Therefore, all of these species in panel III could be detected with these protocols. The primers in protocol 3, 7 and 8 were designed in silico to amplify a segment of the *16S* rRNA gene in most members of the *B*. *burgdorferi s*.*l*. complex and in the relapsing fever borreliae. Laboratory A (protocol 2) did not detect *B*. *japonica* or *B*. *hermsii* when the gene *flaB* was targeted. The reason for this result could be due to mismatches at the primer binding site. The *flaB* gene of *B*. *japonica* has two mismatches within the binding site of the forward primer. Likewise, the *flaB* gene of *B*. *hermsii* has four mismatches at the binding site of the reverse primer, two within the primer sequence and two at the 3’ terminus, and four mismatches at the binding site for the forward primer. However, the PCR assays were probably not designed to detect “relapsing fever” borreliae. Laboratory B (protocol 4) was not able to detect *B*. *spielmanii* PSigII, *B*. *lusitaniae* Poti B2, *B*. *japonica*, *B*. *hermsii* or *B*. *miyamotoi* when the *ospA* gene was targeted. In addition to this, laboratory B (protocol 3) was not able to detect *B*. *lusitaniae* Poti B2 or *B*. *japonica*, in contrast to laboratory D (protocol 7) and laboratory E (protocol 8), which detected *B*. *lusitaniae* Poti B2 as positive using the same set of primer pairs and probes, all amplifying the *16S* rRNA target gene. However, as in protocol 3, these protocols were not able to detect *B*. *japonica* (Table 4 and S4 Table). Further investigation of the binding site of the primers revealed two mismatched bases between the reverse primer and its binding site on the template. One of the two mismatched bases is located at the 3’ terminus and these mismatches may explain the failure of *B*. *japonica* detection by protocols 3, 7 and 8. Regarding the *B*. *lusitaniae* strain, protocols 7 and 8 were both able to detect it even though one mismatched base appears in the probe target sequence. Regarding *B*. *hermsii* and *B*. *miyamotoi*, protocols 3, 7 and 8 should theoretically not be able to detect the two species since there are three mismatched bases and one extra base in the probe targeting sequence. This finding may be a result of the high target gene concentration. However, all three laboratories, B, D and E were able to detect the two species. The unexpected detection of *Leptospira* DNA with protocol 5 may be a result of possible carry-over contamination. In practice, all *16S* PCR-based tests without DNA sequencing of the PCR amplicon for validation are prone to generate this kind of error.

Previous studies have shown that *ospA* is highly variable in European strains and that detection of the gene is dependent on the design of primers and probes [28], which may explain the results for protocol 4. However, it is unclear why some genospecies were not detected by the *16S* rRNA protocols, even though there were theoretically full match at the binding sites for forward and reverse primer for all genospecies, except for the two mismatches in the reverse primer of *B*. *japonica*. This result illustrates the importance of including positive controls, participating in quality programmes and optimization of primer pairs and probes in order to detect newly discovered pathogenic species such as *B*. *spielmanii*.

The principal aim of the study was to compare and evaluate the real-time PCR protocols in the participating laboratories as processes rather than to perform a detailed technical evaluation of effectiveness. Furthermore, the included samples consisted of cultured bacteria of known origin to ensure high quality material for comparison of PCR assays. To further assess the laboratory procedures and interpretation of results in clinical practice and research, it would have been desirable to complement the real-time PCR results with sequencing results.

The data from the present study may contribute to the development of validation criteria for PCR methods for the molecular detection of *B*. *burgdorferi* s.l. in clinical samples in Europe. It is recommended that a PCR method should at least be able to detect *B*. *afzelii*, *B*. *garinii* and *B*. *burgdorferi* with the same sensitivity. However, it is also desirable to have a method that is able to detect other *Borrelia* species such as *B*. *spielmanii*, *B*. *bissetii* and *B*. *lusitaniae*, as human pathogenicity is indicated. By continuous control and optimization of primer and probe specificity it is possible to find new genotypes and mutations which may be highly important.

## Conclusion

In conclusion, we show high analytical sensitivity, analytical specificity and concordance between the eight real-time PCR protocols. Together with the low cross-reactivity for species closely related to *Borrelia*, the findings of this study indicate that the analytical sensitivity problem in CSF samples is not associated with the type of template. To increase the analytical sensitivity, the pre-analytical parts of the methods must be further evaluated and optimized.

## Supporting information

S1 Table Eight PCR protocols for detection of *B*. *burgdorferi* s.l. used in Sweden, Norway and Denmark. The extraction methods are correlated only to panel II and the template volumes to panels I-III.
(DOCX)

S2 Table Ct-value for detection of *B*. *burgdorferi* at five laboratories in Sweden, Norway and Denmark. The values correspond to panel I and are reported as duplicate.
(DOCX)

S3 Table Ct-value for detection of *B*. *burgdorferi* at five laboratories in Sweden, Norway and Denmark. The values correspond to panel II and are reported as duplicate.
(DOCX)

S4 Table Ct-value for detection of *B*. *burgdorferi* at five laboratories in Sweden, Norway and Denmark. The values correspond to panel III and are reported as duplicate.
(DOCX)
