# Supplementary material for: Molecular detection of Borrelia burgdorferi sensu lato – An analytical comparison of real-time PCR protocols from five different Scandinavian laboratories
Source: PLoS One. 2017 Sep 22;12(9):e0185434. doi: 10.1371/journal.pone.0185434 (PMC5609768; doi:10.1371/journal.pone.0185434)
Supplement: S1 Table — The extraction methods are correlated only to panel II and the template volumes to panels I-III. (DOCX) [file pone.0185434.s001.docx]

| **S1 Table:** | | | | | | | | | | |  |  |
| --- | --- | --- | --- | --- | --- | --- | --- | --- | --- | --- | --- | --- |
| **Protocol 1** | | | | | | | | | | | | |
|  | |  |  |  | | |  |  |  |  |  |  |
|  | |  |  |  | | |  |  |  |  |  |  |
| **Extraction method** | | The samples in panel II were centrifuged at 12000 g for 1 h and extraction was performed on the cell pellet in 300 µl cerebrospinal fluid (CSF). The lysis step was performed manually (20 μL Proteinase K) at 56°C for 1 h and the samples was purified using EZ1 RNA Tissue Mini kit (Qiagen, Hilden, Germany) and EZ1 Advanced XL (Qiagen) according to the manufacturer´s instructions. Total nucleic acid (NA) was eluted in a volume of 50 μL RNAse free water. Reverse-transcribed NA (RTNA) synthesis was performed by Illustra ™ Ready-to-Go RT-PCR beads kit as previously described in section “Extraction and reverse-transcription of nucleic acid (panel I)” (GE Healthcare, Amersham, Place, UK) | | | | | | | | | | |
|  | |  |  | |  |  | |  |  |  |  |  |
| **PCR-program** | | Step | Temperature (°C) | | Time (s) | | | |  | | | |
|  | | Activation | 50 | | 120 | | | |  | | | |
|  | | Denaturation | 95 | | 15 | | | |  | | | |
|  | | Anneling | 58 | | 30 | | | |  | | | |
|  | | Extension | 72 | | 30 | | | |  | | | |
|  | |  |  | |  | |  |  |  |  |  |  |
|  | | x45 cycles |  | |  | |  |  |  |  |  |  |
|  | | 95°C for 15 s, followed by cooling to 60°C for 1 min, and subsequent heating to 95°C at 0.8 °C min^–1^ with continuous fluorescence. | | | | |  |  |  |  |  |  |
|  | |  |  |  | | |  |  |  |  |  |  |
| **Mastermix** | | Reagens | Amount (μL) | Final concentration | | | | | Supplier (company) | | | |
|  | | Platinum® qPCR SuperMix UDG | 10 | 1X | | | | | Invitrogen, Carlsbad, US | | | |
|  | | LUX^TM^ Bor16SFL primer (10μM) | 0.4 | 0.2 μM | | | | | TIBMolBiol, Berlin, Germany | | | |
|  | | Unlabelled Bor16SR primer (10 μM) | 0.4 | 0.2 μM | | | | | TIBMolBiol | | | |
|  | | RNAse free water | 4.2 |  | | | | |  | | | |
|  | | Template | 5.0 |  | | | | |  | | | |
|  | |  |  |  | | |  |  |  |  |  |  |
| **Protocol 2** | | | | | | | | | | | | |
|  | |  |  |  | | |  |  |  |  |  |  |
|  | |  |  |  | | |  |  |  |  |  |  |
| **Extraction method** | | The samples in panel II were centrifuged at 12000 g for 1 h and extraction was performed on the cell pellet in 300 µl cerebrospinal fluid (CSF). The lysis step was performed manually (20 μL Proteinase K) at 56°C for 1 h and the samples was purified using EZ1 RNA Tissue Mini kit (Qiagen, Hilden, Germany ) and EZ1 Advanced XL (Qiagen) according to the manufacturer´s instructions. Total nucleic acid (NA) was eluted in a volume of 50 μL RNAse free water. Reverse-transcribed NA (RTNA) synthesis was performed by Illustra ™ Ready-to-Go RT-PCR beads kit as previously described in section “Extraction and reverse-transcription of nucleic acid (panel I)”(GE Healthcare, Amersham, Place, UK) | | | | | | | | | | |
|  | |  |  |  | | | | |  | | | |
| **PCR-program** | | Step | Temperature (°C) | Time (s) | | | | |  | | | |
|  | | Activation | 95 | 600 | | | | |  | | | |
|  | | Denaturation | 95 | 15 | | | | |  | | | |
|  | | Anneling / Extension | 60 | 60 | | | | |  | | | |
|  | |  |  |  | | | | |  | | | |
|  | | x 55 cycle |  |  | | | | |  | | | |
|  | |  |  |  | | | | |  | | | |
| **Mastermix** | | Reagens | Amount (μL) | Final concentration | | | | | Supplier (company) | | | |
|  | | Maxima Probe qPCR master mix (2X) | 12.50 | 1X | | | | | Thermo Fischer Scientific, Waltham, US | | | |
|  | | flaBf (10 μM) | 1.50 | 0.6 μM | | | | | Applied Biosystems Inc., Carlsbad, US | | | |
|  | | flaBr (10 μM) | 1.50 | 0.6 μM | | | | | Applied Biosystems Inc. | | | |
|  | | flaBmA (5 μM) | 1.25 | 0.25 μM | | | | | Applied Biosystems Inc. | | | |
|  | | flaBm3B (5 μM) | 1.25 | 0.25 μM | | | | | Applied Biosystems Inc. | | | |
|  | | ROX (diluted 1:10) | 0.15 |  | | | | | Invitrogen | | | |
|  | | RNase free Water | 1.85 |  | | | | |  | | | |
|  | | Template | 5.00 |  | | | | |  | | | |
|  | |  |  |  | | | | |  | | | |
| **Protocol 3** | | | | | | | | | | | | |
|  | |  |  |  | | | | | |  | | |
|  | |  |  |  | | | | | |  | | |
| **Extraction method** | | The samples in panel II were centrifuged at 12 000 g for 1 h and extraction was performed on the cell pellet in 200 µl CSF. The lysis step was performed manually (Proteinase K) at 56°C for 1 h. The rest of the protocol was performed automatically by QiaCube (Qiagen). | | | | | | | | | | |
|  | |  |  |  | | | | |  | | | |
| **PCR-program** | | Step | Temperature (°C) | Time (s) | | | | |  | | | |
|  | | Preincubation UNG | 40 | 180 | | | | |  | | | |
|  | | Activation | 95 | 600 | | | | |  | | | |
|  | | Denaturation | 95 | 15 | | | | |  | | | |
|  | | Anneling | 60 | 30 | | | | |  | | | |
|  | | Extension | 72 | 20 | | | | |  | | | |
|  | | Cooling | 40 | 20 | | | | |  | | | |
|  | |  |  |  | | | | |  | | | |
|  | | x47 cycles |  |  | | | | |  | | | |
|  | |  |  |  | | | | |  | | | |
| **Mastermix** | | Reagens | Amount (μL) | Final concentration | | | | | Supplier (company) | | | |
|  | | MgCl_2_ | 3.02 | 5 mM | | | | |  | | | |
|  | | LightCycler FastStart DNA master mix 10X | 2.00 | 1X | | | | | Roche Diagnostics, Basel, Switzerland | | | |
|  | | Uracil DNA -glucocylase | 0.50 | 0.5 units | | | | | Eurgentec, Ougrée, Belgium | | | |
|  | | Primers/probe/RNase free water | 9.30 | Primers 0.5 μM and Probe 0.2 μM | | | | | Eurofins Genomics, Ebersberg, Germany /Applied Biosystems Inc. | | | |
|  | | Template | 5.00 |  | | | | |  | | | |
|  | |  |  |  | | | | |  | | | |
| **Protocol 4** | | | | | | | | | | | | |
|  | |  |  |  | | | | | | |  | |
|  | |  |  |  | | | | | | |  | |
| **Extraction method** | | The samples in panel II were centrifuged at 12 000 g for 1 h and extraction was performed on the cell pellet in 200 µl CSF. The lysis step was performed manually (Proteinase K) at 56°C for 1 h. The rest of the protocol was performed automatically by QiaCube (Qiagen). | | | | | | | | | | |
|  | |  |  |  | | | | |  | | | |
| **PCR-program** | | Step | Temperature (°C) | Time (s) | | | | |  | | | |
|  | | Preincubation UNG | 40 | 180 | | | | |  | | | |
|  | | Activation | 95 | 600 | | | | |  | | | |
|  | | Denaturation | 95 | 15 | | | | |  | | | |
|  | | Anneling | 60 | 30 | | | | |  | | | |
|  | | Extension | 72 | 20 | | | | |  | | | |
|  | | Cooling | 40 | 20 | | | | |  | | | |
|  | |  |  |  | | | | |  | | | |
|  | | x47 cycles |  |  | | | | |  | | | |
|  | |  |  |  | | | | |  | | | |
| **Mastermix** | |  |  |  | | | | |  | | | |
|  | | Reagens | Amount (μL) | Final concentration | | | | | Supplier (company) | | | |
|  | | MgCl_2_ | 3.02 | 5 mM | | | | |  | | | |
|  | | LightCycler FastStart DNA master mix 10X | 2.00 | 1X | | | | | Roche Diagnostics | | | |
|  | | Uracil DNA -glucocylase | 0.50 | 0.5 units | | | | | Eurgentec | | | |
|  | | Primers/probe/RNase free water | 9.30 | Primers 0.5 μM and Probe 0.4 μM | | | | | Eurofins Genomics | | | |
|  | | Template | 5.00 |  | | | | |  | | | |
|  | |  |  |  | | | | |  | | | |
| **Protocol 5** | | | | | | | | | | | | |
|  | |  |  |  | | |  |  |  |  |  |  |
|  | |  |  |  | | |  |  |  |  |  |  |
| **Extraction method** | | The samples in panel II were purified using NucliSENS® easyMag® (Biomerieux, Marcy-l'Étoile, France) and NucliSENS® Nucleic Acid Extraction Reagents (Biomerieux) according to manufacturer’s instructions. 500 μL was extracted (no centrifugation) and eluted in a volume of 50 μL. | | | | | | | | | | |
|  | |  |  |  | | | | |  | | | |
| **PCR-program** | | Step | Temperature (°C) | Time (s) | | | | |  | | | |
|  | | Activation | 95° | 600 | | | | |  | | | |
|  | | Denaturation | 95° | 15 | | | | |  | | | |
|  | | Annealing/Extension | 60° | 60 | | | | |  | | | |
|  | |  |  |  | | | | |  | | | |
|  | | x50 cycles |  |  | | | | |  | | | |
|  | |  |  |  | | | | |  | | | |
| **Mastermix** | |  |  |  | | | | |  | | | |
|  | | Reagens | Amount (μL) | Final concentration | | | | | Supplier / Company | | | |
|  | | Brilliant II low ROX (2X) | 12.5 | 1X | | | | | Agilent Technologies Inc., Santa Clara, US | | | |
|  | | 16SBOR_F | 1.00 | 0.4 µM | | | | | Eurofins Genomics | | | |
|  | | 16SBOR_R | 1.00 | 0.4 µM | | | | | Eurofins Genomics | | | |
|  | | 16SBOR_P | 1.00 | 0.1 µM | | | | | Eurofins Genomics | | | |
|  | | RNase free water | 4.50 |  | | | | |  | | | |
|  | | Template | 5.00 |  | | | | |  | | | |
|  | |  |  |  | | | | |  | | | |
| **Protocol 6** | | | | | | | | | | | | |
|  | |  |  |  | | |  |  |  |  |  |  |
|  | |  |  |  | | |  |  |  |  |  |  |
| **Extraction method** | | 200 μL of the samples in panel II were purified using EZ1 DNA Tissue kit (Qiagen) and Biorobot EZ1 (Qiagen) according to manufacturer’s instructions. The samples were eluted in 100 μL. | | | | | | | | | | |
|  | |  |  |  | | | | |  | | | |
| **PCR-program** | | Step | Temperature (°C) | Time (s) | | | | |  | | | |
|  | | Pre-PCR | 95 | 180 | | | | |  | | | |
|  | | Denaturation | 95 | 10 | | | | |  | | | |
|  | | Anneling / extension | 60 | 50 | | | | |  | | | |
|  | |  |  |  | | | | |  | | | |
|  | | x50 cycles |  |  | | | | |  | | | |
|  | |  |  |  | | | | |  | | | |
| **Mastermix** | |  |  |  | | | | |  | | | |
|  | | Reagens | Amount (μL) | Final concentration | | | | | Supplier / Company | | | |
|  | | SensiFAST Probe No-ROX Kit | 12.5 | 1x | | | | | Bioline, London, England | | | |
|  | | 16SBOR-Fw |  | 0.4 µM | | | | | Eurofins Genomics | | | |
|  | | 16SBOR-Rev |  | 0.4 µM | | | | | Eurofins Genomics | | | |
|  | | 16SBOR-P |  | 0.1 µM | | | | | Eurofins Genomics | | | |
|  | | Template | 5.0 |  | | | | |  | | | |
|  | |  |  |  | | | | |  | | | |
| **Protocol 7** | | | | | | | | | | | | |
|  | |  |  |  | | |  |  |  |  |  |  |
|  | |  |  |  | | |  |  |  |  |  |  |
| **Extraction method** | | 200 μL of the samples in panel II were purified using EZ1 DNA Tissue kit (Qiagen) and Biorobot EZ1 (Qiagen) according to manufacturer´s instructions. The samples were eluted in 100 μL. | | | | | | | | | | |
|  | |  |  |  | | | | |  | | | |
| **PCR-program** | | Step | Temperature (°C) | Time (s) | | | | |  | | | |
|  | | Denaturation | 95 | 10 | | | | |  | | | |
|  | | Anneling / Extension | 60 | 50 | | | | |  | | | |
|  | |  |  |  | | | | |  | | | |
|  | | x50 cycles |  |  | | | | |  | | | |
|  | |  |  |  | | | | |  | | | |
| **Mastermix** | |  |  |  | | | | |  | | | |
|  | | Reagens | Amount (μL) | Final concentration | | | | | Supplier / Company | | | |
|  | | SensiFAST Probe No-ROX Kit | 12.5 | 1x | | | | | Bioline | | | |
|  | | 16S Bor-sp-Fw |  | 0.900 µM | | | | | Eurofins Genomics | | | |
|  | | 16SBOR-Rev |  | 0.900 µM | | | | | Eurofins Genomics | | | |
|  | | 16SBor-sp-P |  | 0.225 µM | | | | | Life Technologies | | | |
|  | | Templat | 5.0 |  | | | | |  | | | |
|  | |  |  |  | | | | |  | | | |
| **Protocol 8** | | | | | | | | | | | | |
|  | |  |  |  | | | | | | |  | |
|  | |  |  |  | | | | | | |  | |
| **Extraction method** | | Protocol 8 was not included in reference panel II. | | | | | | | | | | |
|  | |  |  |  | | | | |  | | | |
| **PCR-program** | | Step | Temperature (°C) | Time (s) | | | | |  | | | |
|  | | Preincubation UNG | 50 | 120 | | | | |  | | | |
|  | | Activation | 95 | 600 | | | | |  | | | |
|  | | Denaturation | 94 | 30 | | | | |  | | | |
|  | | Anneling | 53 | 30 | | | | |  | | | |
|  | | Extension | 72 | 30 | | | | |  | | | |
|  | |  |  |  | | | | |  | | | |
|  | | x55 cycles |  |  | | | | |  | | | |
|  | |  |  |  | | | | |  | | | |
| **Mastermix** | |  |  |  | | | | |  | | | |
|  | | Reagens | Amount (μL) | Final concentration | | | | | Supplier / Company | | | |
|  | | TaqMan Universal PCR Master Mix (2X) | 10 | 1X | | | | | Applied Biosystems Inc. | | | |
|  | | 16S F (100 µM) | 0.25 | 1.25 µM | | | | | Applied Biosystems Inc. | | | |
|  | | 16S R (100 µM) | 0.25 | 1.25 µM | | | | | Applied Biosystems Inc. | | | |
|  | | LD-probe (100 µM) | 0.05 | 0.25 µM | | | | | Applied Biosystems Inc. | | | |
|  | | ddH2O | 7.45 |  | | | | |  | | | |
|  | | Template | 5.00 |  | | | | |  | | | |
