# Supplementary material for: Molecular detection of Borrelia burgdorferi sensu lato – An analytical comparison of real-time PCR protocols from five different Scandinavian laboratories
Source: PLoS One. 2017 Sep 22;12(9):e0185434. doi: 10.1371/journal.pone.0185434 (PMC5609768; doi:10.1371/journal.pone.0185434)
Supplement: S2 Table — The values correspond to panel I and are reported as duplicate. (DOCX) [file pone.0185434.s002.docx]

|  |  |  |  | **Laboratory A** | |  | **Laboratory B** | |  | **Laboratory C** |  | **Laboratory D** | |  | **Laboratory E** |
| --- | --- | --- | --- | --- | --- | --- | --- | --- | --- | --- | --- | --- | --- | --- | --- |
|  |  |  |  | ***16S* rRNA** | ***flaB*** |  | ***16S* rRNA** | ***ospA*** |  | ***16S* rRNA** |  | ***16S* rRNA** | ***16S* rRNA** |  | ***16S* rRNA** |
|  |  | **Strain** | **Concentration** | **Protocol 1** | **Protocol 2** |  | **Protocol 3** | **Protocol 4** |  | **Protocol 5** |  | **Protocol 6** | **Protocol 7** |  | **Protocol 8** |
|  |  | *B. afzelii* Lu81 | 10^4 | 26;26 | 32;32 |  | 27;27 | 34;34 |  | 29;29 |  | 27;27 | 31;31 |  | 27;25 |
|  |  |  | 10^3 | 31;31 | 36;34 |  | 31;31 | 37;36 |  | 32;32 |  | 32;32 | 35;35 |  | 30;30 |
|  |  |  | 10^2 | 36;35 | n.d.;n.d. |  | 34;33 | n.d.;39 |  | 36;37 |  | 37;37 | 38;39 |  | 34;34 |
|  |  |  | 10^1 | 40;39 | n.d.;n.d. |  | 37;37 | n.d.;n.d. |  | 43;41 |  | 41;42 | 41;41 |  | 38;38 |
|  |  |  | 10^0 | n.d. | n.d.;n.d. |  | 38;n.d. | n.d.;n.d. |  | n.d.;n.d. |  | n.d.;n.d. | n.d.;n.d. |  | n.d.;n.d. |
|  |  | *B. garinii* Lu59 | 10^4 | 25;25 | 30;29 |  | 26;27 | 30;30 |  | 28;28 |  | 26;26 | 30;30 |  | 24;25 |
|  |  |  | 10^3 | 29;29 | 33;32 |  | 29;30 | 32;32 |  | 30;30 |  | 29;29 | 33;33 |  | 27;27 |
|  |  |  | 10^2 | 32;33 | 37;37 |  | 33;34 | 37;37 |  | 34;34 |  | 34;34 | 37;37 |  | 32;32 |
|  |  |  | 10^1 | 35;35 | 37;37 |  | 35;34 | 38;n.d. |  | 36;36 |  | 36;36 | 39;38 |  | 34;34 |
|  |  |  | 10^0 | n.d.;n.d. | n.d.;n.d. |  | 39;n.d. | n.d.;n.d. |  | n.d.;n.d. |  | n.d.;n.d. | n.d.;n.d. |  | n.d.;n.d. |
|  |  | *B.burgdorferi s.s.* B31 | 10^4 | 27;27 | 34;33 |  | 28;27 | 36;37 |  | 28;28 |  | 28;28 | 31;31 |  | 25;26 |
|  |  |  | 10^3 | 30;30 | 38;36 |  | 29;29 | 40;41 |  | 31;31 |  | 31;31 | 34;34 |  | 29;29 |
|  |  |  | 10^2 | 36;36 | n.d.;n.d. |  | 34;34 | n.d.;n.d. |  | 37;37 |  | 37;37 | 39;39 |  | 34;34 |
|  |  |  | 10^1 | 39;39 | n.d.;n.d. |  | 36;37 | n.d.;n.d. |  | 40;40 |  | 40;40 | 41;41 |  | 36;36 |
|  |  |  | 10^0 | 42;41 | n.d.;n.d. |  | 38;n.d. | n.d.;n.d. |  | n.d.;n.d. |  | n.d.;n.d. | n.d.;n.d. |  | 38;n.d. |
|  |  | *E. coli* J1 | 50.0 | n.d.;n.d. | n.d.;n.d. |  | n.d.;n.d. | n.d.;n.d. |  | n.d.;n.d. |  | n.d.;n.d. | n.d.;n.d. |  | n.d.;n.d. |
|  |  |  | 50.0 | n.d.;n.d. | n.d.;n.d. |  | n.d.;n.d. | n.d.;n.d. |  | n.d.;n.d. |  | n.d.;n.d. | n.d.;n.d. |  | n.d.;n.d. |
|  |  | RNase-free water |  | n.d.;n.d. | n.d.;n.d. |  | n.d.;n.d. | n.d.;n.d. |  | n.d.;n.d. |  | n.d.;n.d. | n.d.;n.d. |  | n.d.;n.d. |
|  |  |  |  | n.d.;n.d. | n.d.;n.d. |  | n.d.;n.d. | n.d.;n.d. |  | n.d.;n.d. |  | n.d.;n.d. | n.d.;n.d. |  | n.d.;n.d. |
|  |  |  |  | n.d.;n.d. | n.d.;n.d. |  | n.d.;n.d. | n.d.;n.d. |  | n.d.;n.d. |  | n.d.;n.d. | n.d.;n.d. |  | n.d.;n.d. |
|  |  | n.d. = not detected |  |  |  |  |  |  |  |  |  |  |  |  |  |

**S2 Table:**
