# Supplementary material for: Molecular detection of Borrelia burgdorferi sensu lato – An analytical comparison of real-time PCR protocols from five different Scandinavian laboratories
Source: PLoS One. 2017 Sep 22;12(9):e0185434. doi: 10.1371/journal.pone.0185434 (PMC5609768; doi:10.1371/journal.pone.0185434)
Supplement: S3 Table — The values correspond to panel II and are reported as duplicate. (DOCX) [file pone.0185434.s003.docx]

|  |  | **Laboratory A** | |  | **Laboratory B** | |  | **Laboratory C** |  | **Laboratory D** | |  |  |  |  |
| --- | --- | --- | --- | --- | --- | --- | --- | --- | --- | --- | --- | --- | --- | --- | --- |
|  |  | **cDNA** | **cDNA** |  | **DNA** | **DNA** |  | **DNA** |  | **DNA** | **DNA** |  |  |  |  |
| **Strain** | **Concentration** | **Protocol 1** | **Protocol 2** |  | **Protocol 3** | **Protocol 4** |  | **Protocol 5** |  | **Protocol 6** | **Protocol 7** |  |  |  |  |
| *B. afzelii* Lu81 | 10^4 | 36;36 | 38:37:00 |  | 35;35 | 32;32 |  | 33;33 |  | 34;34 | 36;36 |  |  |  |  |
|  | 10^3 | 40;41 | 42:40:00 |  | 35;34 | 33;32 |  | 36;36 |  | 37;38 | 38;39 |  |  |  |  |
|  | 10^2 | n.d.;n.d. | n.d.;n.d. |  | 38;38 | 38.0;n.d. |  | 38;40 |  | 39;40 | 39;40 |  |  |  |  |
|  | 10^1 | n.d.;n.d. | n.d.;n.d. |  | n.d.;40 | 38.0;n.d. |  | n.d.;n.d. |  | n.d.;n.d. | n.d.;n.d. |  |  |  |  |
|  | 10^0 | n.d.;n.d. | n.d.;n.d. |  | n.d.;n.d. | n.d.;n.d. |  | n.d.;n.d. |  | n.d.;n.d. | n.d.;n.d. |  |  |  |  |
| *B. garinii* Lu59 | 10^4 | 34;34 | 35;32 |  | 33;33 | 30:30:00 |  | 32;31 |  | 31;31 | 34;34 |  |  |  |  |
|  | 10^3 | 38;38 | 41;38 |  | 36;36 | 33:33:00 |  | 35;34 |  | 35;36 | 37;37 |  |  |  |  |
|  | 10^2 | 42;40 | 43;42 |  | 36;38 | 36:35:00 |  | 38;38 |  | 38;42 | 40;40 |  |  |  |  |
|  | 10^1 | n.d.;n.d. | n.d.;n.d. |  | n.d.;n.d. | n.d.;n.d. |  | 40;42 |  | 42;40 | 40;n.d. |  |  |  |  |
|  | 10^0 | n.d.;n.d. | n.d.;n.d. |  | 39;n.d. | 38.0;n.d. |  | n.d.;n.d. |  | 41;42 | n.d.;40 |  |  |  |  |
| *B.burgdorferi s.s.* B31 | 10^4 | 34;34 | 41;42 |  | 32;32 | 31;30 |  | 33;32 |  | 33;33 | 35;35 |  |  |  |  |
|  | 10^3 | 37;37 | 40;39 |  | 35;36 | 35;35 |  | 35;35 |  | 35;35 | 38;38 |  |  |  |  |
|  | 10^2 | n.d.;n.d. | 52;n.d. |  | 38;38 | 36;38 |  | 39;40 |  | 41;n.d. | 41;n.d. |  |  |  |  |
|  | 10^1 | n.d.;n.d. | n.d.;n.d. |  | n.d.;n.d. | n.d.;n.d. |  | n.d.;n.d. |  | n.d.;n.d. | n.d.;n.d. |  |  |  |  |
|  | 10^0 | n.d.;n.d. | n.d.;n.d. |  | n.d.;n.d. | n.d.;n.d. |  | n.d.;n.d. |  | n.d.;n.d. | n.d.;n.d. |  |  |  |  |
| n.d. = not detected |  |  |  |  |  |  |  |  |  |  |  |  |  |  |  |

**S3 Table:**
