# Supplementary material for: Molecular detection of Borrelia burgdorferi sensu lato – An analytical comparison of real-time PCR protocols from five different Scandinavian laboratories
Source: PLoS One. 2017 Sep 22;12(9):e0185434. doi: 10.1371/journal.pone.0185434 (PMC5609768; doi:10.1371/journal.pone.0185434)
Supplement: S4 Table — The values correspond to panel III and are reported as duplicate. (DOCX) [file pone.0185434.s004.docx]

**S4 Table:**

|  |  | **Laboratory A** | |  | **Laboratory B** | |  | **Laboratory C** |  | **Laboratory D** | |  | **Laboratory E** |
| --- | --- | --- | --- | --- | --- | --- | --- | --- | --- | --- | --- | --- | --- |
| **Strain** | **Concentration** | **Protocol 1** | **Protocol 2** |  | **Protocol 3** | **Protocol 4** |  | **Protocol 5** |  | **Protocol 6** | **Protocol 7** |  | **Protocol 8** |
| *B. burgdorferi sensu stricto* B31 | 10^4 | 29;29 | 23;23 |  | 26;24 | 23;24 |  | 27;n.a. |  | 23;n.a. | 27;n.a. |  | 23;23 |
|  | 10^3 | 33;32 | 27;27 |  | 30;29 | 27;30 |  | 30;n.a. |  | 26;n.a. | 30;n.a. |  | 26;26 |
|  | 10^2 | 37;37 | 30;31 |  | 33;32 | 30;32 |  | 34;n.a. |  | 30;n.a. | 33;n.a. |  | 30;30 |
|  | 10^1 | 43;42 | 33;35 |  | 35;35 | 33;35 |  | 37;n.a. |  | 34;n.a. | 36;n.a. |  | 33;33 |
|  | 10^0 | n.d.;44 | 39;38 |  | 37;36 | 37;42 |  | 41;n.a. |  | 41;n.a. | 39;n.a. |  | 39;36 |
|  | 10^-1 | n.d.;n.d. | n.d.;39 |  | n.d.;n.d. | 41;n.d. |  | 40;n.a. |  | 43;n.a. | n.d.;n.a. |  | 40;38 |
| *B. burgdorferi sensu stricto* Pbre | 10^4 | 29;28 | 24;24 |  | 27;25 | 24;25 |  | 28;n.a. |  | 23;n.a. | 27;n.a. |  | 24;24 |
|  | 10^3 | 33;33 | 27;28 |  | 30;29 | 27;29 |  | 31;n.a. |  | 27;n.a. | 31;n.a. |  | 27;27 |
|  | 10^2 | 38;37 | 31;31 |  | 33;32 | 31;33 |  | 35;n.a. |  | 31;n.a. | 33;n.a. |  | 30;31 |
|  | 10^1 | 41;40 | 35;35 |  | 35;35 | 33;36 |  | 37;n.a. |  | 35;n.a. | 36;n.a. |  | 34;34 |
|  | 10^0 | n.d.;n.d. | 36;37 |  | 37;36 | 36;39 |  | 40;n.a. |  | n.d.;n.a. | 39;n.a. |  | 37;37 |
|  | 10^-1 | n.d.;n.d. | n.d.;n.d. |  | n.d.;n.d. | n.d.;n.d. |  | 40;n.a. |  | n.d.;n.a. | n.d.;n.a. |  | n.d.;n.d. |
| *B. afzelii* Pko | 10^4 | 29;28 | 22;22 |  | 26;25 | 21;21 |  | 27;n.a. |  | 23;n.a. | 26;n.a. |  | 23;22 |
|  | 10^3 | 33;33 | 25;26 |  | 29;28 | 25;25 |  | 31;n.a. |  | 26;n.a. | 30;n.a. |  | 26;26 |
|  | 10^2 | 37;37 | 29;29 |  | 33;31 | 28;28 |  | 33;n.a. |  | 30;n.a. | 33;n.a. |  | 30;30 |
|  | 10^1 | 41;39 | 33;33 |  | 34;34 | 31;32 |  | 36;n.a. |  | 33;n.a. | 36;n.a. |  | 33;34 |
|  | 10^0 | n.d.;n.d. | 36;37 |  | 38;37 | 34;36 |  | 40;n.a. |  | 38;n.a. | 38;n.a. |  | 36;37 |
|  | 10^-1 | n.d.;n.d. | n.d.;n.d. |  | 39;38 | 36;39 |  | 40;n.a. |  | n.d.;n.a. | 40;n.a. |  | n.d.;n.d. |
| *B. afzelii* PVPM | 10^4 | 29;29 | 22;22 |  | 27;26 | 23;24 |  | 27;n.a. |  | 22;n.a. | 26;n.a. |  | 23;23 |
|  | 10^3 | 33;33 | 26;26 |  | 30;29 | 26;27 |  | 30;n.a. |  | 27;n.a. | 30;n.a. |  | 27;27 |
|  | 10^2 | 37;36 | 29;30 |  | 33;31 | 29;29 |  | 34;n.a. |  | 30;n.a. | 33;n.a. |  | 30;30 |
|  | 10^1 | 42;41 | 33;33 |  | 36;34 | 32;34 |  | 36;n.a. |  | 35;n.a. | 36;n.a. |  | 33;34 |
|  | 10^0 | n.d.;n.d. | 37;36 |  | 36;37 | 34;37 |  | 40;n.a. |  | 39;n.a. | 39;n.a. |  | 37;38 |
|  | 10^-1 | n.d.;n.d. | 38;39 |  | n.d.;40 | n.d.;41 |  | 42;n.a. |  | n.d.;n.a. | n.d.;n.a. |  | n.d.;n.d. |
| *B. garinii* PBr | 10^4 | 29;29 | 23;23 |  | 27;26 | 23;25 |  | 27;n.a. |  | 23;n.a. | 27;n.a. |  | 24;24 |
|  | 10^3 | 34;34 | 26;26 |  | 31;29 | 27;27 |  | 31;n.a. |  | 28;n.a. | 30;n.a. |  | 27;27 |
|  | 10^2 | 38;38 | 30;30 |  | 33;32 | 29;30 |  | 34;n.a. |  | 31;n.a. | 34;n.a. |  | 31;31 |
|  | 10^1 | 44;44 | 33;33 |  | 36;35 | 32;34 |  | 38;n.a. |  | 35;n.a. | 37;n.a. |  | 34;34 |
|  | 10^0 | 45;45 | 37;37 |  | 40;37 | 36;40 |  | 39;n.a. |  | 40;n.a. | n.d.;n.a. |  | 39;38 |
|  | 10^-1 | n.d.;n.d. | n.d.;n.d. |  | 38;40 | n.d.;39 |  | 42;n.a. |  | 41;n.a. | n.d.;n.a. |  | n.d.;n.d. |
| *B. garinii* Phei | 10^4 | 31;31 | 24;24 |  | 28;27 | 24;23 |  | 29;n.a. |  | 25;n.a. | 28;n.a. |  | 25;25 |
|  | 10^3 | 35;34 | 27;28 |  | 32;31 | 27;30 |  | 32;n.a. |  | 28;n.a. | 31;n.a. |  | 28;28 |
|  | 10^2 | 39;38 | 31;31 |  | 34;33 | 30;31 |  | 35;n.a. |  | 33;n.a. | 35;n.a. |  | 32;31 |
|  | 10^1 | 44;44 | 35;34 |  | 36;36 | 33;35 |  | 38;n.a. |  | 37;n.a. | 37;n.a. |  | 34;35 |
|  | 10^0 | n.d.;n.d. | n.d.;n.d. |  | 40;37 | 36;38 |  | 41;n.a. |  | 40;n.a. | 39;n.a. |  | 39;37 |
|  | 10^-1 | n.d.;n.d. | n.d.;n.d. |  | n.d.;n.d. | 38;n.d. |  | 0;n.a. |  | n.d.;n.a. | n.d.;n.a. |  | n.d.;n.d. |
| *B. garinii* PwudII | 10^4 | 29;28 | 23;23 |  | 27;26 | 23;25 |  | 27;n.a. |  | 23;n.a. | 27;n.a. |  | 24;24 |
|  | 10^3 | 33;33 | 26;27 |  | 28;28 | 28;28 |  | 31;n.a. |  | 27;n.a. | 30;n.a. |  | 27;27 |
|  | 10^2 | 38;38 | 30;30 |  | 33;32 | 29;30 |  | 34;n.a. |  | 31;n.a. | 34;n.a. |  | 30;31 |
|  | 10^1 | 43;41 | 33;34 |  | 35;35 | 33;34 |  | 38;n.a. |  | 35;n.a. | 36;n.a. |  | 34;34 |
|  | 10^0 | n.d.;n.d. | n.d.;37 |  | 39;37 | 36;40 |  | 43;n.a. |  | 40;n.a. | 39;n.a. |  | 39;38 |
|  | 10^-1 | n.d.;n.d. | n.d.;n.d. |  | n.d.;38 | n.d.;39 |  | 41;n.a. |  | n.d.;n.a. | n.d.;n.a. |  | n.d.;n.d. |
| *B. garinii* Pref | 10^4 | 30;29 | 23;24 |  | 27;27 | 23;23 |  | 29;n.a. |  | 24;n.a. | 28;n.a. |  | 24;24 |
|  | 10^3 | 34;34 | 27;27 |  | 31;30 | 26;27 |  | 32;n.a. |  | 27;n.a. | 31;n.a. |  | 27;28 |
|  | 10^2 | 38;38 | 30;31 |  | 34;33 | 31;33 |  | 34;n.a. |  | 31;n.a. | 34;n.a. |  | 31;31 |
|  | 10^1 | 44;42 | 34;33 |  | 36;36 | 34;35 |  | 38;n.a. |  | 36;n.a. | 37;n.a. |  | 35;34 |
|  | 10^0 | n.d.;n.d. | 39;38 |  | 38;37 | 36;38 |  | 40;n.a. |  | 41;n.a. | 39;n.a. |  | 36;38 |
|  | 10^-1 | n.d.;n.d. | n.d.;n.d. |  | n.d.;n.d. | 38;40 |  | n.d.;n.a. |  | 43;n.a. | 40;n.a. |  | 43;n.d. |
| *B. garinii* Pla | 10^4 | 31;30 | 24;24 |  | 29;27 | 26;30 |  | 29;n.a. |  | 24;n.a. | 28;n.a. |  | 25;25 |
|  | 10^3 | 35;34 | 27;28 |  | 31;30 | 29;32 |  | 32;n.a. |  | 28;n.a. | 31;n.a. |  | 28;28 |
|  | 10^2 | 39;39 | 31;31 |  | 34;33 | 32;36 |  | 35;n.a. |  | 32;n.a. | 35;n.a. |  | 31;32 |
|  | 10^1 | 44;45 | 34;34 |  | 37;36 | 35;41 |  | 40;n.a. |  | 37;n.a. | 37;n.a. |  | 34;34 |
|  | 10^0 | 45;44 | 38;38 |  | 37;38 | 40;n.d. |  | 42;n.a. |  | 41;n.a. | n.d.;n.a. |  | 49;37 |
|  | 10^-1 | n.d.;n.d. | n.d.;n.d. |  | n.d.;n.d. | n.d.;n.d. |  | n.d.;n.a. |  | 41;n.a. | n.d.;n.a. |  | 47;39 |
| *B. spielmanii* PSigII | 10^4 | 31;31 | 24;24 |  | 29;28 | n.d.;n.d. |  | 29;n.a. |  | 25;n.a. | 29;n.a. |  | 25;25 |
|  | 10^3 | 35;34 | 28;28 |  | 31;31 | n.d.;n.d. |  | 32;n.a. |  | 29;n.a. | 32;n.a. |  | 28;28 |
|  | 10^2 | 40;39 | 32;32 |  | 35;34 | n.d.;n.d. |  | 35;n.a. |  | 34;n.a. | 36;n.a. |  | 32;32 |
|  | 10^1 | 44;42 | 35;36 |  | 37;35 | n.d.;n.d. |  | 40;n.a. |  | 37;n.a. | 38;n.a. |  | n.d.;36 |
|  | 10^0 | n.d.;n.d. | n.d.;n.d. |  | n.d.;37 | n.d.;n.d. |  | 42;n.a. |  | 40;n.a. | 40;n.a. |  | 37;39 |
|  | 10^-1 | n.d.;n.d. | n.d.;42 |  | n.d.;n.d. | n.d.;n.d. |  | n.d.;n.a. |  | n.d.;n.a. | n.d.;n.a. |  | n.d.;n.d. |
| *B. bavariensis* Pbi | 10^4 | 31;31 | 25;25 |  | 28;27 | 23;23 |  | 29;n.a. |  | 25;n.a. | 28;n.a. |  | 25;25 |
|  | 10^3 | 36;35 | 29;29 |  | 31;30 | 27;27 |  | 32;n.a. |  | 29;n.a. | 32;n.a. |  | 28;28 |
|  | 10^2 | 39;39 | 32;32 |  | 35;34 | 30;32 |  | 35;n.a. |  | 32;n.a. | 35;n.a. |  | 32;32 |
|  | 10^1 | 45;44 | 36;36 |  | 37;35 | 33;34 |  | 39;n.a. |  | 36;n.a. | 38;n.a. |  | 34;35 |
|  | 10^0 | n.d.;n.d. | 38;37 |  | n.d.;37 | 36;38 |  | 42;n.a. |  | 40;n.a. | 40;n.a. |  | n.d.;n.d. |
|  | 10^-1 | n.d.;n.d. | n.d.;n.d. |  | n.d.;n.d. | n.d.;n.d. |  | 0;n.a. |  | n.d.;n.a. | n.d.;n.a. |  | n.d.;n.d. |
| *B. bissetti* PGeb | 10^4 | 28;28 | 23;23 |  | 26;25 | 25;n.d. |  | 27;n.a. |  | 22;n.a. | 27;n.a. |  | 23;23 |
|  | 10^3 | 34;34 | 26;26 |  | 30;29 | 28;27 |  | 30;n.a. |  | 27;n.a. | 30;n.a. |  | 27;27 |
|  | 10^2 | 37;37 | 30;30 |  | 32;31 | 30;33 |  | 34;n.a. |  | 31;n.a. | 33;n.a. |  | 30;30 |
|  | 10^1 | 41;41 | 33;33 |  | 36;34 | 34;n.d. |  | 37;n.a. |  | 34;n.a. | 36;n.a. |  | 33;34 |
|  | 10^0 | n.d.;n.d. | 37;35 |  | 37;36 | 37;n.d. |  | 40;n.a. |  | n.d.;n.a. | 37;n.a. |  | 37;37 |
|  | 10^-1 | n.d.;n.d. | n.d.;n.d. |  | 0;0 | 40;n.d. |  | 40;n.a. |  | n.d.;n.a. | n.d.;n.a. |  | 38;n.d. |
| *B. lusitaniae* Poti B2 | 10^4 | 28;28 | 26;27 |  | n.d.;n.d. | n.d.;n.d. |  | 27;n.a. |  | 23;n.a. | 33;n.a. |  | 22;23 |
|  | 10^3 | 35;32 | 30;30 |  | n.d.;n.d. | n.d.;n.d. |  | 29;n.a. |  | 27;n.a. | 36;n.a. |  | 26;26 |
|  | 10^2 | 36;35 | 34;34 |  | n.d.;n.d. | n.d.;n.d. |  | 33;n.a. |  | 31;n.a. | 39;n.a. |  | 30;30 |
|  | 10^1 | 40;39 | 38;38 |  | n.d.;n.d. | n.d.;n.d. |  | 37;n.a. |  | 34;n.a. | 43;n.a. |  | 33;33 |
|  | 10^0 | n.d.;n.d. | 40;39 |  | n.d.;n.d. | n.d.;n.d. |  | 41;n.a. |  | 39;n.a. | 46;n.a. |  | 37;36 |
|  | 10^-1 | n.d.;n.d. | n.d.;42 |  | n.d.;n.d. | n.d.;n.d. |  | 42;n.a. |  | n.d.;n.a. | 45;n.a. |  | n.d.;39 |
| *B. valaisiana* VS116 | 10^4 | 30;30 | 23;24 |  | 30;28 | 23;22 |  | 28;n.a. |  | 24;n.a. | 29;n.a. |  | 25;25 |
|  | 10^3 | 34;34 | 27;27 |  | 33;32 | 27;28 |  | 31;n.a. |  | 28;n.a. | 33;n.a. |  | 28;29 |
|  | 10^2 | 38;38 | 31;31 |  | 35;34 | 30;30 |  | 34;n.a. |  | 32;n.a. | 35;n.a. |  | 32;32 |
|  | 10^1 | 41;43 | 35;35 |  | 37;36 | 33;33 |  | 38;n.a. |  | 36;n.a. | 38;n.a. |  | 35;35 |
|  | 10^0 | n.d.;n.d. | 39;37 |  | 40;39 | n.d.;40 |  | 42;n.a. |  | 42;n.a. | 40;n.a. |  | 37;40 |
|  | 10^-1 | n.d.;n.d. | 38;n.d. |  | 38;38 | 38;n.d. |  | n.d.;n.a. |  | 41;n.a. | n.d.;n.a. |  | n.d.;n.d. |
| *B. japonica* | 10^4 | 33;33 | n.d.;n.d. |  | n.d.;n.d. | n.d.;n.d. |  | 29;n.a. |  | 25;n.a. | n.d.;n.a. |  | n.d.;n.d. |
|  | 10^3 | 37;37 | n.d.;n.d. |  | n.d.;n.d. | n.d.;n.d. |  | 32;n.a. |  | 30;n.a. | n.d.;n.a. |  | n.d.;n.d. |
|  | 10^2 | 41;41 | n.d.;n.d. |  | n.d.;n.d. | n.d.;n.d. |  | 36;n.a. |  | 33;n.a. | n.d.;n.a. |  | n.d.;n.d. |
|  | 10^1 | n.d.;n.d. | n.d.;n.d. |  | n.d.;n.d. | n.d.;n.d. |  | 39;n.a. |  | 37;n.a. | n.d.;n.a. |  | 43;n.d. |
|  | 10^0 | n.d.;n.d. | n.d.;n.d. |  | n.d.;44 | n.d.;n.d. |  | 42;n.a. |  | n.d.;n.a. | n.d.;n.a. |  | n.d.;n.d. |
|  | 10^-1 | n.d.;n.d. | n.d.;n.d. |  | n.d.;n.d. | n.d.;n.d. |  | n.d.;n.a. |  | n.d.;n.a. | n.d.;n.a. |  | n.d.;n.d. |
| *B. hermsii* | 10^4 | 33;33 | n.d.;n.d. |  | 38;39 | n.d.;n.d. |  | 30;n.a. |  | 27;n.a. | 40;n.a. |  | 29;30 |
| *B. miyamotoi* | 10^4 | 29;29 | 45;44 |  | 38;35 | n.d.;n.d. |  | 27;n.a. |  | 23;n.a. | 36;n.a. |  | 26;26 |
| *T. phagedenis* | 10^4 | n.d.;n.d. | n.d.;n.d. |  | n.d.;n.d. | n.d.;n.d. |  | n.d.;n.a. |  | n.d.;n.a. | n.d.;n.a. |  | n.d.;n.d. |
| *Leptospiren* | 10^4 | n.d.;n.d. | n.d.;n.d. |  | n.d.;n.d. | n.d.;n.d. |  | 43;n.a. |  | n.d.;n.a. | n.d.;n.a. |  | n.d.;n.d. |
| *Leptospiren* | 10^4 | n.d.;n.d. | n.d.;n.d. |  | n.d.;n.d. | n.d.;41 |  | n.d.;n.a. |  | n.d.;n.a. | n.d.;n.a. |  | n.d.;n.d. |

| \| n.d. = not detected \|  \|  \|  \|  \|  \|  \|  \|  \|  \|  \|  \|  \|  \| \| --- \| --- \| --- \| --- \| --- \| --- \| --- \| --- \| --- \| --- \| --- \| --- \| --- \| --- \| \| n.a. = not analysed. The samples marked with n.a. was analysed as single samples. \|  \|  \|  \|  \|  \|  \|  \|  \|  \|  \|  \|  \|  \| |
| --- | --- | --- | --- | --- | --- | --- | --- | --- | --- | --- | --- | --- | --- | --- | --- | --- | --- | --- | --- | --- | --- | --- | --- | --- | --- | --- | --- | --- |
|  |
